# The Effect of Maternal Immune Activation on Social Play-Induced Ultrasonic Vocalization in Rats

**DOI:** 10.3390/brainsci11030344

**Published:** 2021-03-09

**Authors:** Kinga Gzielo, Agnieszka Potasiewicz, Ewa Litwa, Diana Piotrowska, Piotr Popik, Agnieszka Nikiforuk

**Affiliations:** Maj Institute of Pharmacology, Polish Academy of Sciences, Department of Behavioral Neuroscience and Drug Development, 12 Smętna Street, 31-343 Kraków, Poland; gzielo@if-pan.krakow.pl (K.G.); potasiew@if-pan.krakow.pl (A.P.); litwa@if-pan.krakow.pl (E.L.); dpiotrow@if-pan.krakow.pl (D.P.); nfpopik@cyf-kr.edu.pl (P.P.)

**Keywords:** ultrasonic vocalization, maternal immune activation, autism, communication, rat, sex differences, social play, rats

## Abstract

Prenatal maternal infection is associated with an increased risk of various neurodevelopmental disorders, including autism spectrum disorders (ASD). Maternal immune activation (MIA) can be experimentally induced by prenatal administration of polyinosinic:polycytidylic acid (poly I:C), a synthetic viral-like double-stranded RNA. Although this MIA model is adopted in many studies, social and communicative deficits, included in the first diagnostic criterion of ASD, are poorly described in the offspring of poly(I:C)-exposed dams. This study aimed to characterize the impact of prenatal poly(I:C) exposure on socio-communicative behaviors in adolescent rats. For this purpose, social play behavior was assessed in both males and females. We also analyzed quantitative and structural changes in ultrasonic vocalizations (USVs) emitted by rats during the play test. Deficits of social play behaviors were evident only in male rats. Males also emitted a significantly decreased number of USVs during social encounters. Prenatal poly(I:C) exposure also affected acoustic call parameters, as reflected by the increased peak frequencies. Additionally, repetitive behaviors were demonstrated in autistic-like animals regardless of sex. This study demonstrates that prenatal poly(I:C) exposure impairs socio-communicative functioning in adolescent rats. USVs may be a useful tool for identifying early autistic-like abnormalities.

## 1. Introduction

Environmental conditions during prenatal life can influence fetal brain development. One of the most well-known factors linked to neurodevelopmental disorders is infection [1]. Plenty of studies and meta-analyses of human data have shown that maternal infection increases the risk of neuropsychiatric disorders in children [2]. The neurodevelopmental consequences of maternal immune activation (MIA) have also been confirmed in animal models [3]. One of the most frequently applied experimental MIA protocols is based on prenatal exposure of pregnant dams to polyinosinic:polycytidylic acid (poly(I:C)) [4]. Poly(I:C) is a synthetic analog of double-stranded RNA that mimics a viral infection. This MIA-based model has been widely used to explore the biochemical, neuroanatomical, and behavioral aspects of neuropsychiatric conditions such as autism spectrum disorders (ASD) [5].

ASD is a heterogeneous neurodevelopmental disorder that is considered one of the most severe public health problems because of its early onset, lifelong persistence, and significant limitations in everyday functioning [6]. According to DSM-5 (Diagnostic and Statistical Manual of Mental Disorders), the ASD symptoms are grouped into two main diagnostic criteria that are social/communicative deficits and restricted, repetitive patterns of behaviors, interests, or activities [7]. One of the most troublesome aspects of ASD is that the number of patients has strikingly increased in the last years [8]. Despite the severity of the problem, no specific and effective drug treatment is currently available for patients diagnosed with ASD, particularly in the social/communication domain [9]. Therefore, there is a need for preclinical research to better understand ASD etiopathology and to develop novel pharmacotherapeutic strategies.

Social and communicative deficits, included in the first diagnostic criterion of ASD, can also be modeled in preclinical paradigms [10]. Rodents, especially rats, are very social animals with a highly developed pattern of social behaviors, which are directly related to conspecifics [11]. Social play behavior is the first non-mother-directed social behavior in rats [12]. It emerges three weeks after birth and peaks between the 28th and 40th postnatal days. It is crucial to study social play behavior precisely during this period because some of the specific playful events decline when the animals become sexually mature. Playful interactions can be split into socio-positive behaviors such as grooming, pinning, pouncing, sniffing, and socio-negative behaviors, which are mostly associated with aggression, such as boxing, avoiding, and pushing away the conspecific [13,14].

During the rough-and-tumble play, rats also communicate using ultrasonic vocalizations (USVs) [15,16]. The USVs can be divided into two basic groups, such as 22-kHz “unhappy” calls and 50-kHz “happy” calls. The low frequency (22-kHz) calls, termed alarms, are emitted in response to unpleasant events, e.g., during losing aggressive encounters, facing predators, or experiencing anxiety. On the other hand, ultrasounds at about 50-kHz are associated with rewarding events and encourage and boost playing [15]. These “happy” calls are heterogeneous and may differ in pattern and duration [17]. They comprise flat (i.e., constant frequency) and frequency modulated ultrasounds. The frequency modulated calls are further divided into several categories, including “trills” that appear in spectrograms as rhythmic waves of ups and downs and are considered the most characteristic type of frequency-modulated calls. Thus, the rich repertoire of rats’ juvenile play behaviors accompanied by a complex acoustic communication system may be employed to model early-onset socio-communicative deficits.

This study characterized poly(I:C)-induced socio-communicative deficits in early adolescence. To this aim, we analyzed social play behavior and recorded USVs emitted by juvenile rats during the social play test. We performed the detailed characteristics of the acoustic calls’ features that provide a more comprehensive assessment of rats’ USVs than by using purely quantitative measures. To the best of our knowledge, there is little data on ASD-related social behaviors during the adolescent period in a poly(I:C) model (detailed description in Section 4). None of the studies examined the potential outcome of poly(I:C) on rats’ juvenile play. Even less is known about the impact of MIA on ultrasonic communication, and most existing studies are restricted to the assessment of neonatal USVs [18]. Based on previous studies using other rat ASD models [19,20], we hypothesized that juvenile play deficits and impaired vocalization should also occur due to poly(I:C) treatment.

To study another core symptom of ASD, i.e., repetitive behaviors, we employed a marble-burying test that is commonly used to score repetitive digging behavior [21]. Additionally, the number of repetitive/stereotypic-like movements was measured using activity meters.

Autism spectrum disorders affect males more frequently than females [22]. However, sex-specific differences in the manifestation of autistic features may lead to delayed or missed diagnoses in women [23]. It has also been recently shown that women are better at concealing their autism [24]. Nevertheless, the male prevalence in ASD is also reflected in animal studies that have been predominantly focused on males while often excluding females. Consequently, our understanding of sex-differential vulnerability to ASD may be incomplete. Therefore, we chose to compare the two sexes in this study.

## 2. Materials and Methods

### 2.1. Animals

Pregnant dams (Sprague-Dawley rats, *N* = 18) were obtained from Charles River (Sulzfeld, Germany) on gestation day (GD) 9–10. They were housed individually in polycarbonate cages: 26.5 (width) × 18 (height) × 42 (length) cm. On postnatal day (PND) 21, pups were weaned and separated by sex and litter into groups of 3–5 rats. Females and males were housed in different temperature-controlled (21 ± 1 °C) and humidity-controlled (40–50%) colony rooms under a 12/12 h light/dark cycle (lights on at 06:00 h). Food and water were available ad libitum. Behavioral testing was performed during the light phase of the light/dark cycle. The experiments were conducted in accordance with the European Guidelines for animal welfare (2010/63/EU) and were approved by the II Local Ethics Committee for Animal Experiments at the Maj Institute of Pharmacology, Polish Academy of Science, Krakow, Poland.

### 2.2. Poly(I:C) Administration

On GD 15, the dams were injected intraperitoneally (i.p.) with either physiological saline (vehicle) (*N* = 9) or poly(I:C) at a dose of 5 mg/kg (*N* = 9). Poly(I:C) (Sigma-Aldrich, Poznan, Poland) was dissolved in physiological saline. Both poly(I:C) and vehicle were administered at a volume of 2 mL/kg. The dose and time of administration of poly(I:C) were based on previous reports demonstrating autistic-like behaviors, including social and communicative abnormalities [25,26,27]. There were no effects of treatment on gestation length and litter size (average number ≈ 12), but there was a prevalence of females in poly(I:C) offspring (male/female ratio: ≈50:50 and ≈40:60 for control and poly(I:C) groups, respectively). The rats prenatally exposed to poly(I:C) appeared healthy and could not be physically or behaviorally distinguished from the controls. They also did not differ from control animals in body weight (Figure S1, Table S1).

In total, 64 males and 54 females were born from 9 vehicle-treated dams and 39 males and 60 females from 9 poly(I:C)-treated dams. In order to minimize the risk of a litter effect, offspring were randomly distributed across the experimental procedure, and none of the animals was tested more than twice. Detailed group characteristics are provided in the given method description.

### 2.3. Social Play Test (PND 32–35)

The test procedure was conducted in same-sex, same-treatment pairs, as previously described [20,28]. One day before the test, the rats were transported to the experimental room, weighed, and the backsides of one-half of the animals were marked with a Pentel permanent marker. Next, they were individually adapted to the test area for about 5 min. The dimly illuminated (15 Lux) test area consisted of a rectangular, polycarbonate cage (width × height × length: 38 × 20 × 59 cm) with approximately 2 cm of wood shavings covering the floor. On the testing day, each rat was isolated in a non-transparent plastic cage (width × height × length: 22 × 15 × 28 cm) for 2.5 h before the test. Then, two unfamiliar (various cages/litters) rats of matched body weights (±5 g) were placed in the test area, and their behaviors were recorded for 10 min using the Observer software (Noldus Information Technology, Wageningen, The Netherlands). After the test, the rats were returned to their home cages. The testing arena was subsequently emptied of wood shavings and thoroughly cleaned with water before being refilled with fresh bedding.

The social play behavior of each rat was separately analyzed by an experienced observer blind to the experimental conditions using the Observer software. We scored the number of episodes of pouncing (sniffing of the conspecific’s neck, followed by rubbing movement) and pinning (upon contact with the nape, the recipient animal fully rotates to a supine position while the other subject stands over it) that were considered the main indices of social play behavior in rats [14]. Based on these scores, play responsiveness was calculated as the percentage of responses (being pinned) to play solicitation (pouncing). Additionally, the total play duration represented the summed time of pouncing, pinning, and play-wrestling behaviors.

Since each animal in a pair yielded similar social behavior scores, the results are expressed as the summed score of each pair of animals. The numbers of pairs used in the analysis were: *N* = 17 (vehicle males), *N* = 16 (vehicle females), *N* = 11 (poly(I:C) males), *N* = 18 (poly(I:C) females). Two pairs (poly(I:C) males) were excluded from the analysis due to failure in the recording.

### 2.4. USV Recording

As previously described [20,28], the rats’ vocalizations were recorded during the entire test session (i.e., 10 min) using a frequency response range of 2 kHz–200 kHz microphone (UltraSoundGate Condensor Microphone CM16/CMPA, Avisoft Bioacoustics, Berlin, Germany) suspended 25 cm above the floor of the test area. Microphone signals were fed into an UltraSoundGate 416H (Avisoft Bioacoustics, Berlin, Germany) before the analog signal was digitized with a sampling rate of 200 kHz and a 16-bit resolution. Acoustic data were recorded using Raven Pro Interactive Sound Analysis Software, version 1.5 (The Cornell Lab of Ornithology Bioacoustics Research Program, Ithaca, NY, USA). The calls were manually marked on the computer screen and counted by an experienced user, blind to the treatment, using the Raven Pro software (The Cornell Lab of Ornithology Bioacoustics Research Program, Ithaca, NY, USA). The spectrograms were generated with a fast Fourier transform (FFT)-length of 512 points and a time-window overlap of 75% (100% frame, Hamming window).

We analyzed: (a) the number of 50-kHz USVs (expressed as a total number of USVs emitted by a pair of rats) and the following 50-kHz USV features: (b) the peak frequency (the frequency in kHz at which maximal energy occurs within the selection, (c) the call duration (length of the call, measured in milliseconds), and (d) the bandwidth (the difference between the highest and lowest frequencies, a measure of frequency modulation, expressed in kHz). We also manually divided the calls (based on their acoustic call features) into the following general types: short calls, flat calls with a near-constant frequency, and frequency-modulated calls. The frequency-modulated calls were subsequently classified as trills, one-component calls (complex, ramp, and inverted-U calls), and multi-component calls (multi-step, step-up, step-down, and composite calls) [20,29]. Moreover, call subtypes were visualized using Kernel density plots depicting peak frequency versus call duration. The 22-kHz alarms were excluded from the analysis due to their negligible distribution (0–1.9%).

### 2.5. Locomotor and Repetitive/Stereotypic-Like Activity (PND 35–38)

Spontaneous locomotor activity was measured automatically in Opto-Varimex-4 Auto-Tracks (Columbus Instruments, Columbus, OH, USA) located in the sound attenuated and ventilated boxes. The Auto-Track System sensed the motion with a grid of infrared photocells (16 beams per *x*- and *y*-axis) surrounding the arena. The data collected every 5 min during a 30-min session are presented as the total distance traveled. Moreover, the number of repetitive/stereotypic-like movements was defined as the number of repeated breaks of the same beam. The number of animals in a given group was: *N* = 20 (vehicle males), *N* = 18 (vehicle females), *N* = 13 (poly(I:C) males), *N* = 22 (poly(I:C) females).

### 2.6. Marble-Burying Test (PND 35–38)

Clean cages (27 × 16.5 × 12.5 cm) were filled with a 4-cm layer of chipped wood bedding. Twenty-five green glass marbles (20 mm diameter) were gently laid on top of the bedding, equidistant from each other in a 5 × 5 arrangement. Animals were placed into the testing cage, and the number of marbles buried (>50% marble covered by bedding material) in 30 min was recorded. Additionally, the distance traveled was automatically measured using the Any-maze^®^ tracking system (Stoelting Co., Wood Dale, IL, USA). The number of animals in a given group was: *N* = 19 (vehicle males), *N* = 18 (vehicle females), *N* = 13 (poly(I:C) males), *N* = 22 (poly(I:C) females).

### 2.7. Statistics

Data on social play behavior (i.e., pouncing episodes and total play duration), USV number, and acoustic call features (peak frequency, duration, and bandwidth) were analyzed by two-way ANOVAs with treatment (vehicle vs. poly(I:C)) and sex (male vs. female) as the between-subject factors. When there was a significant main effect of treatment or sex, we used the Tukey HSD post hoc test to assess overall differences between treatment or sex conditions. The Student’s *t*-test was used for the planned comparisons between vehicle and poly(I:C) treatment conditions within a given sex.

In case data were not normally distributed (i.e., pinning episodes, latency to pinning, play responsiveness, and marble-burying test), differences between groups were analyzed using the Mann–Whitney U-test.

Data on the percentage distribution of call categories were arcsine-transformed and subjected to a repeated measure ANOVA with treatment and sex as between-subject factors and call type as a repeated measure. The Newman–Keuls multiple comparison test was used to analyze between-group differences in call type distribution. Locomotor activity data were analyzed by repeated measure ANOVAs with treatment and sex as between-subject factors and measurement time as a repeated measure. The sphericity was verified using Mauchly’s test. If the assumption of sphericity was rejected, the corrected Greenhouse–Geisser value was utilized.

The effect size was estimated using partial eta squared (ŋ_p_^2^). The normality of data distribution was evaluated by the Kolmogorov-Smirnov test. Statistical significance was set at *p* < 0.05. The statistical analyses were performed using Statistica 12.0 for Windows (StatSoft Inc, Tulsa, OK, USA).

## 3. Results

### 3.1. Social Play Behavior

**Figure 1:**
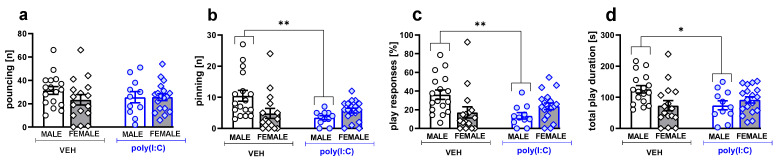
Poly (I:C) exposure reduced social play behavior in males. Data are presented as a mean ± SEM of the number of pouncing (**a**) and pinning episodes (**b**), the percentage of play responsiveness (**c**), and total play duration (**d**). Symbols: ** *p* < 0.01, * *p* < 0.05, a significant difference between vehicle- and poly(I:C)-exposed animals in a given sex group (Mann–Whitney U test (**b**,**c**) and Student’s *t*-test (**d**)).

While poly(I:C) animals did not differ from the vehicle-treated controls in the number of pouncing episodes (Figure 1a, Table S2), the treatment sex-dependently affected pinning behavior (Figure 1b,c). The number of pinning episodes (U = 25, *p* = 0.0015, Mann–Whitney U test, Figure 1b) and play responsiveness (U = 28, *p* = 0.0022, Mann–Whitney U test, Figure 1c) were reduced in poly(I:C) males compared to their controls. Moreover, the latency to pinning was increased in poly(I:C) males (U = 43, *p* = 0.019; Mann–Whitney U test, vehicle male: 172 ± 28 s, poly(I:C) male: 334 ± 526 s, vehicle female: 371 ± 46 s, poly(I:C) female: 295 ± 43 s). Poly(I:C) exposure also reduced total play durations in male rats (*t* = 2.773, *p* = 0.012, Student’s *t*-test; ANOVA treatment × sex interaction: F(1,58) = 6.95, *p* = 0.011, Figure 1d, Table S2).

### 3.2. Ultrasonic Vocalizations

**Figure 2:**
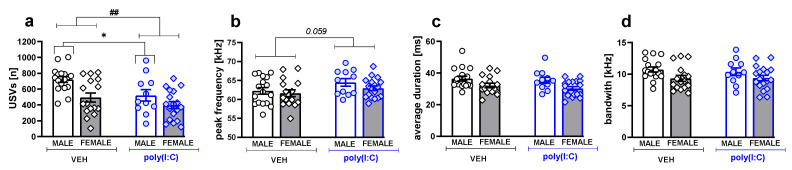
Poly (I:C) exposure decreased the number of USVs and changed their acoustic characteristics. Data are presented as a mean ± SEM of the total number (**a**), peak frequency (**b**), average duration (**c**), and bandwidth (**d**) of the calls. Symbols: ^##^ *p* < 0.01, an overall difference between vehicle- and poly(I:C)-exposed animals (Tukey HSD post hoc test following a significant treatment effect), * *p* < 0.05, a significant difference between vehicle- and poly(I:C)-exposed animals in a given sex group (Student’s *t*-test).

Poly(I:C) exposure also affected USV emission during social play. However, this effect was not sex-specific, as ANOVA analysis revealed significant effects of treatment on the number of USVs (F(1,58) = 8.32, *p* = 0.005, Figure 2a, Table S2) and peak frequency (F(1,58) = 4.32, *p* = 0.042, Figure 2b, Table S2), but insignificant treatment × sex interactions (Table S2). Poly(I:C)-exposed animals emitted a lower number of calls compared to the controls (*p* = 0.002, Tukey post hoc test following a significant treatment effect, Figure 2a, Table S2), and they tended to emit calls of a higher peak frequency (*p* = 0.059, Tukey post hoc test following a significant treatment effect, Figure 2b, Table S2). However, between-treatment comparisons within each sex group revealed a significant reduction only for the USV number in males (*t* = 2.705, *p* = 0.012, Student’s *t*-test). There was no effect of poly(I:C) treatment on other acoustic parameters of calls, i.e., duration (Figure 2c, Table S2) and bandwidth (Figure 2d, Table S2).

Regardless of the treatment, females emitted less USVs (*p* = 0.0003, Tukey HSD post hoc test following a significant sex effect: F(1,58) = 11.49, *p* = 0.0013; Figure 2a, Table S2) and their calls were shorter (*p* = 0.002, Tukey HSD post hoc test following a significant sex effect: F(1,58) = 10.12, *p* = 0.002; Figure 2c, Table S2) and of a narrower frequency bandwidth (*p* = 0.011, Tukey HSD post hoc test following a significant sex effect: F(1,58) = 6.28, *p* = 0.015; Figure 2d, Table S2).

**Figure 3:**
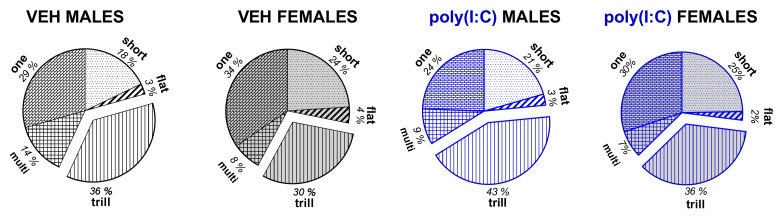
Poly(I:C) changed the percentage distribution of call categories within the tested groups. There was a significant overall difference between vehicle- and poly(I:C)-exposed animals in the distribution of trill calls (*p* < 0.05, Newman–Keuls post hoc test following a significant treatment × call category interaction).

*Call type characteristics*. By analyzing the call type distribution, we observed that regardless of sex, poly(I:C) rats produced a higher proportion of trill calls (*p* = 0.014, Newman–Keuls post hoc test following a significant treatment × call type interaction: F(4,232) = 2.963, *p* = 0.021; Figure 3, Table S2). Analysis of individual call types revealed that the trills emitted by poly(I:C)-treated rats did not differ from those emitted by their controls in any of the acoustic parameters measured (Figure S2, Table S3). Between-treatment comparisons within each sex group did not reveal any significant differences in call distributions in either males or females.

Moreover, females emitted more of the short (*p* = 0.013) and one-component calls (*p* = 0.023), but fewer trills (*p* = 0.006) and multi-component calls (*p* = 0.036, Newman–Keuls post hoc test following a significant sex × call type interaction: F(4,232) = 5.04, *p* = 0.0007; Figure 3, Table S2).

**Figure 4:**
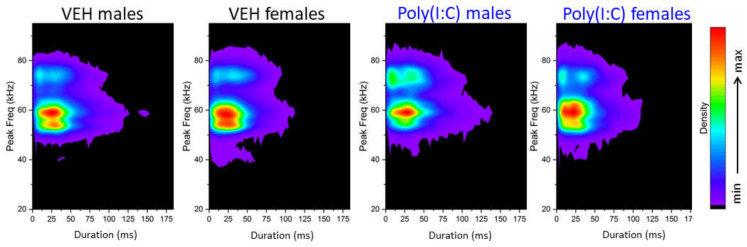
Density plots illustrating the distribution of individual calls depending on their peak frequencies versus durations in vehicle- and poly(I:C)-exposed rats. The densities were visualized in a color-coded way. Poly(I:C) did not change the overall USV cluster distributions, but an enhanced representation of high-frequency calls was observed for poly(I:C) animals.

*Density plots*. To identify clusters of USVs emitted by vehicle and poly(I:C) animals, we used density plots illustrating the distribution of individual calls depending on their peak frequencies versus call durations. Visual inspections showed that poly(I:C) did not affect the overall distribution of USVs (Figure 4). However, an enhanced representation of high-frequency calls in poly(I:C) animals corroborated the peak frequency increases as shown in Figure 2.

### 3.3. Locomotor Activity and Stereotypic-Like Movements

**Figure 5:**
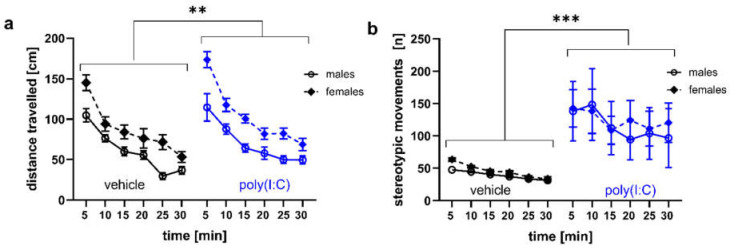
Poly(I:C) exposure elevated locomotor activity and stereotypic-like movements. Data are presented as a mean ± SEM of the distance traveled (**a**) and stereotypic-like movements (**b**) assessed during a 30-min test session. Symbols: ** *p* < 0.01, *** *p* < 0.001: a significant overall difference between vehicle- and poly(I:C)-exposed animals (Tukey HSD post hoc test following a significant treatment effect).

Irrespective of sex, poly(I:C) exposure enhanced rats’ locomotor activity (*p* = 0.0012, Tukey HSD post hoc test following a significant treatment effect: F(1,69) = 5.91, *p* = 0.018; Figure 5a, Table S2) and increased the number of stereotypic movements (*p* = 0.0006, Tukey HSD post hoc test following a significant treatment effect: F(1,69) = 12.47, *p* = 0.0007; Figure 5b, Table S2). Moreover, there was an overall effect of sex on locomotor activity, as the distance traveled was increased in females (*p* = 0.0001, Tukey HSD post hoc test following a significant sex effect: F(1,69) = 5.91, *p* = 0.012; Figure 5a, Table S2).

### 3.4. Marble-Burying

**Figure 6:**
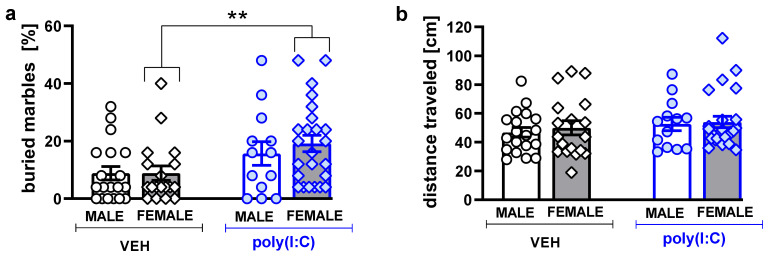
Poly(I:C) exposure increased repetitive-like marble-burying behavior. Data are presented as the percentage of buried marbles (**a**) and distance traveled (**b**). Symbol: ** *p* < 0.01, a significant difference between vehicle- and poly(I:C)-exposed animals in a given sex group (Mann–Whitney U test).

Poly(I:C) exposure significantly increased the number of buried marbles in females (U = 110, *p* = 0.0054, Mann–Whitney U test, Figure 6a). There was no effect of treatment on the distance traveled during the test session (Figure 6b, Table S2), suggesting that changes in locomotor activity were not a confounding factor when scoring buried marbles.

## 4. Discussion

This study demonstrates that prenatal poly(I:C) exposure impairs socio-communicative functioning in adolescent rats. Deficits of social play behaviors were evident only in male rats. While poly(I:C) treatment reduced USV emission during the social encounter irrespectively of sex, significant reductions were demonstrated only in males. Acoustic call parameters were also slightly affected, as reflected by the increased peak frequencies of the emitted calls. Additionally, repetitive behaviors were demonstrated in autistic-like animals, regardless of sex.

Prenatal exposition to poly(I:C) altered the pattern of social play behavior in males. While poly(I:C) males did not differ from controls in play-soliciting behaviors (i.e., pouncing), their responsiveness to play solicitation was decreased, as reflected by the reduced frequency of pinning and increased latency to pinning. To the best of our knowledge, no studies have examined the impact of prenatal poly(I:C) administration on juvenile rats’ social play. Most of the published reports in this area have utilized the three-chamber test, which provides an easily quantifiable sociability measure reflected as a tendency to spend more time in the compartment with an unfamiliar conspecific than in the empty compartment. In line with the social deficits observed in the current experiments, poly(I:C) exposure affected sociability in adolescent [30,31] or adult [32,33] mice and in rats during early adulthood [34] (but see also [35,36] for the opposite results). The above-cited research reports were mainly carried out on males. Females were included in only a few studies demonstrating reductions of females’ sociability assessed in a three-chamber test [25,31] or no effect [36,37]. However, deficits in juvenile rats’ play behavior were noted in another MIA model based on administering the bacterial endotoxin lipopolysaccharide (LPS) [38]. Interestingly, prenatal LPS exposure reduced the frequency of juvenile play behavior exclusively in males [39]. Similar sex-specific impairments were observed in the current poly(I:C) studies that may corroborate the higher prevalence of ASD in males. It has been recently suggested that these protective effects may be attributed to sex differences in immune responses [40]. Accordingly, pro-inflammatory factors were increased in both sexes, but anti-inflammatory factors were decreased in males and increased in females.

However, considering the well-described sexual dimorphism in rough-and-tumble play, it is not surprising that females engage in fewer play behaviors than males. Consequently, a floor effect due to a relatively low level of playful interactions in females may not allow for the demonstration of further decrements. In line with this assumption, social deficits in other behavioral measures were noted in poly(I:C) females [25,31]. Interestingly, our previous study, demonstrating vocalization deficits in valproic acid (VPA)-exposed females [20], suggested that USV assessment in females may be a more sensitive measure of autistic-like disturbances than other indices of juvenile social behavior. Likewise, poly(I:C) treatment reduced USV emission during the social encounter irrespectively of sex (a significant treatment effect in the absence of sex × treatment interaction). However, significant reductions were demonstrated only in males (between-group comparisons), suggesting that communicative deficits in poly(I:C) females may be less pronounced than those previously demonstrated in VPA females.

The demonstrated vocalization deficit in poly(I:C)-exposed rats is per se interesting as there is a limited number of data on USVs in MIA models, and most of them focus on isolation-induced USVs in pups. Several studies have reported decreased USV emission in rat and mouse pups from poly(I:C)-exposed mothers, but others also noted enhanced isolation-induced vocalization (reviewed in [18]). The observed vocalization deficits did not result from altered maternal care [41]. While these discrepancies may arise from diverse poly(I:C) administration schedules, one may conclude that early communication abnormalities occur in this model. Less is known about MIA effects on vocalization during the latter stages of development. For example, adult male rats prenatally exposed to poly(I:C) emitted an increased number of aversive 22-kHz USVs during fear conditioning [26] that further supports the link between immune activation and socio-emotional communication. Moreover, adult poly(I:C) male mice emitted fewer calls during social interactions with either males or females [32,33]. However, other reports did not observe poly(I:C) effects on male mice’s USV emission during direct interactions with females [35] or when exposed to female urine [40]. While we are unaware of any studies concerned with juvenile USVs in the poly(I:C) model, social play-induced USVs were decreased in LPS exposed male offspring [38]. The effects of LPS on juvenile social play behavior were also assessed by the recording of USVs emitted in response to manual tickling by an experimenter [42]. However, the latter study demonstrated only greater USV variability in LPS-exposed adolescent male and female rats [42]. The literature mentioned above, together with the present findings, indicate that vocalization abnormalities occur in MIA models. Nevertheless, the paucity of female data hinders a definite conclusion on sex differences in this measure.

There are limited data available on the qualitative analysis of USVs in MIA models. For example, calls emitted by poly(I:C) exposed mice in response to a social encounter were characterized by a shortened average duration [32]. However, this parameter was not affected when analyzing social play-evoked USVs in LPS-treated rats [38]. While we also did not observe significant differences in call durations between poly(I:C)- and vehicle-exposed animals, poly(I:C) exposure increased USV peak frequencies. Interestingly, poly(I:C) exposed pups also emitted calls of an elevated frequency when separated from their mothers [27]. Similarly, our previous study employing a valproic acid model of ASD demonstrated higher frequency calls in neonatal and adolescent rats [20]. Hence, it is tempting to interpret these atypical call features as a signature of autistic-like ultrasonic communication.

Moreover, call type categorizations revealed that poly(I:C) mice produced significantly fewer two-step frequency and chevron calls than controls [33]. The changes in call distribution were also observed in our study, as poly(I:C) rats emitted an increased proportion of trills. Interestingly, we found a similar tendency in VPA-exposed rats [20]. Considering that trills prevail before playful actions [43,44], their increased percentage may reflect the enhanced anticipatory aspects of social play. Indeed, poly(I:C) rats seem to be equally motivated as controls to initiate play, as revealed by unchanged play soliciting behaviors, but their reduced play responsiveness may suggest that their trills do not efficiently stimulate play behavior. In line with this assumption, we analyzed the acoustic feature of trills; however, in contrast to previous findings in a VPA model, no differences were seen between poly(I:C) and control rats. Alternatively, altered distribution of USV subtypes may result from an aberrant organization of social behaviors. Indeed, the temporal analysis of USVs emitted during social play revealed a close connection between specific call categories and particular play events [45]. Unfortunately, the lack of the temporal correlation analysis between the USV call sequence and specific behavior limits our ability to draw definitive conclusions regarding the current study. Finally, the trill emission may also be related to general arousal [46], which would agree with the increased locomotor activity in poly(I:C) rats.

The neurobehavioral origin of the USV deficit may be related to the altered processing of sensory information. Impaired processing and integration of auditory information is common in autistic children and may lead to delayed language development [47]. Interestingly, deficient sensory processing has also been reported in MIA models [25]. Besides, a growing body of evidence has shown that alterations in the periaqueductal gray, a brainstem nucleus engaged in the production of vocalizations and indirect modulation (via motor neurons in the brainstem) of vocal fold vibration, may be the leading cause of impaired communication in mammals [48,49]. Further studies are necessary to answer whether sensory or motor dysfunctions lead to USV alterations and consequently which brain regions (ascending auditory pathway or the periaqueductal gray) are involved in the MIA-induced vocal communication impairment.

Besides social and communication deficits, ASD symptoms include restricted, repetitive, and stereotyped patterns of behavior. Common motor stereotypies displayed by rodents are repetitive digging behaviors that are assessed in the marble-burying test. In line with previously published data [32,33], poly(I:C) exposure increased the number of buried marbles. However, it should be noticed that the higher number of marbles buried by the poly(I:C) offspring may not necessarily result from goal-directed digging behavior but, for example, from enhanced circling behavior (Figure S3). Possible alterations in anxiety levels in poly(I:C)-exposed animals (e.g., [32]) may also affect the outcome of this test. Nevertheless, the locomotor pattern of poly(I:C) rats was also characterized by stereotypic-like movements.

In contrast to social deficits, repetitive behaviors were exhibited to a greater extent in females. Similarly, VPA prenatal exposure induced autistic-like behaviors in a sex-specific manner [50]. While females were less susceptible to VPA-evoked socio-emotional deficits than males, they also demonstrated stereotypic behavior [50]. These results further support the necessity of the inclusion of females in ASD studies.

## 5. Conclusions

The current study demonstrated that the poly(I:C)-based model recapitulates ASD-related behavioral abnormalities. Similar to human ASD patients, affected offspring exhibit social/communicative impairments and repetitive behaviors. The finding that vocalization deficits occur in the poly(I:C)-based MIA model supports the utility of USVs as a measure of autistic-like socio-communicative abnormalities. The question of whether these changes persist beyond the adolescent period requires further study.

## Data Availability

Data are contained within the article and Supplementary Materials.

## Supplementary Materials

The following are available online at https://www.mdpi.com/2076-3425/11/3/344/s1, Figure S1. Body weight, Figure S2. The acoustic characteristics of trill calls, Figure S3. Poly (I:C) treatment increased circling behaviors in females, Table S1. Body weight—ANOVA results, Table S2. The results of the ANOVA analysis, Table S3. The acoustic characteristics of trill calls—ANOVA results.

## References

[1] Scola G., Duong A. (2017). Prenatal maternal immune activation and brain development with relevance to psychiatric disorders. Neuroscience.

[2] Estes M.L., McAllister A.K. (2015). Immune mediators in the brain and peripheral tissues in autism spectrum disorder. Nat. Rev. Neurosci..

[3] Ji-Xu A., Vincent A. (2020). Maternal Immunity in Autism Spectrum Disorders: Questions of Causality, Validity, and Specificity. J. Clin. Med..

[4] Meyer U. (2014). Prenatal Poly(I:C) Exposure and Other Developmental Immune Activation Models in Rodent Systems. Biol. Psychiatry.

[5] Haddad F.L., Patel S.V., Schmid S. (2020). Maternal Immune Activation by Poly I:C as a preclinical Model for Neurodevelopmental Disorders: A focus on Autism and Schizophrenia. Neurosci. Biobehav. Rev..

[6] Lord C., Brugha T.S., Charman T., Cusack J., Dumas G., Frazier T., Jones E.J.H., Jones R.M., Pickles A., State M.W. (2020). Autism spectrum disorder. Nat. Rev. Dis. Primers.

[7] Association A.P. (2013). Diagnostic and Statistical Manual of Mental Disorders (DSM-5^®^).

[8] Christensen D.L., Maenner M.J., Bilder D., Constantino J.N., Daniels J., Durkin M.S., Fitzgerald R.T., Kurzius-Spencer M., Pettygrove S.D., Robinson C. (2019). Prevalence and Characteristics of Autism Spectrum Disorder Among Children Aged 4 Years—Early Autism and Developmental Disabilities Monitoring Network, Seven Sites, United States, 2010, 2012, and 2014. MMWR Surveill. Summ..

[9] LeClerc S., Easley D. (2015). Pharmacological therapies for autism spectrum disorder: A review. Pharm. Ther..

[10] Wohr M., Scattoni M.L. (2013). Behavioural methods used in rodent models of autism spectrum disorders: Current standards and new developments. Behav. Brain Res..

[11] Schweinfurth M.K. (2020). The social life of Norway rats (*Rattus norvegicus*). eLife.

[12] Panksepp J. (1981). The ontogeny of play in rats. Dev. Psychobiol..

[13] Vanderschuren L.J.M.J., Trezza V., Andersen S.L., Pine D.S. (2014). What the Laboratory Rat has Taught us about Social Play Behavior: Role in Behavioral Development and Neural Mechanisms. The Neurobiology of Childhood.

[14] Vanderschuren L.J.M.J., Achterberg E.J.M., Trezza V. (2016). The neurobiology of social play and its rewarding value in rats. Neurosci. Biobehav. Rev..

[15] Pellis S.M., Burke C.J., Kisko T.M., Euston D.R., Brudzynski S.M. (2018). Chapter 11—50-kHz Vocalizations, Play and the Development of Social Competence. Handbook of Behavioral Neuroscience.

[16] Simola N., Brudzynski S.M., Brudzynski S.M. (2018). Chapter 17—Repertoire and Biological Function of Ultrasonic Vocalizations in Adolescent and Adult Rats. Handbook of Behavioral Neuroscience.

[17] Wright J.M., Gourdon J.C., Clarke P.B.S. (2010). Identification of multiple call categories within the rich repertoire of adult rat 50-kHz ultrasonic vocalizations: Effects of amphetamine and social context. Psychopharmacology.

[18] Jouda J., Wohr M., Del Rey A. (2019). Immunity and ultrasonic vocalization in rodents. Ann. N. Y. Acad. Sci..

[19] Raza S., Himmler B.T., Himmler S.M., Harker A., Kolb B., Pellis S.M., Gibb R. (2015). Effects of prenatal exposure to valproic acid on the development of juvenile-typical social play in rats. Behav. Pharmacol..

[20] Gzielo K., Potasiewicz A., Hołuj M., Litwa E., Popik P., Nikiforuk A. (2020). Valproic acid exposure impairs ultrasonic communication in infant, adolescent and adult rats. Eur. Neuropsychopharmacol..

[21] Thomas A., Burant A., Bui N., Graham D., Yuva-Paylor L.A., Paylor R. (2009). Marble burying reflects a repetitive and perseverative behavior more than novelty-induced anxiety. Psychopharmacology.

[22] Loomes R., Hull L., Mandy W.P.L. (2017). What Is the Male-to-Female Ratio in Autism Spectrum Disorder? A Systematic Review and Meta-Analysis. J. Am. Acad Child. Adolesc. Psychiatry.

[23] Lai M.-C., Baron-Cohen S. (2015). Identifying the lost generation of adults with autism spectrum conditions. Lancet Psychiatry.

[24] Hull L., Lai M.-C., Baron-Cohen S., Allison C., Smith P., Petrides K.V., Mandy W. (2019). Gender differences in self-reported camouflaging in autistic and non-autistic adults. Autism.

[25] Lins B.R., Marks W.N., Zabder N.K., Greba Q., Howland J.G. (2019). Maternal Immune Activation during Pregnancy Alters the Behavior Profile of Female Offspring of Sprague Dawley Rats. eNeuro.

[26] Yee N., Schwarting R.K., Fuchs E., Wohr M. (2012). Increased affective ultrasonic communication during fear learning in adult male rats exposed to maternal immune activation. J. Psychiatr. Res..

[27] Potasiewicz A., Gzielo K., Popik P., Nikiforuk A. (2020). Effects of prenatal exposure to valproic acid or poly(I:C) on ultrasonic vocalizations in rat pups: The role of social cues. Physiol. Behav..

[28] Potasiewicz A., Holuj M., Piotrowska D., Zajda K., Wojcik M., Popik P., Nikiforuk A. (2019). Evaluation of ultrasonic vocalizations in a neurodevelopmental model of schizophrenia during the early life stages of rats. Neuropharmacology.

[29] Golebiowska J., Hołuj M., Potasiewicz A., Piotrowska D., Kuziak A., Popik P., Homberg J.R., Nikiforuk A. (2019). Serotonin transporter deficiency alters socioemotional ultrasonic communication in rats. Sci. Rep..

[30] Vuillermot S., Luan W., Meyer U., Eyles D. (2017). Vitamin D treatment during pregnancy prevents autism-related phenotypes in a mouse model of maternal immune activation. Mol. Autism.

[31] Aavani T., Rana S.A., Hawkes R., Pittman Q.J. (2015). Maternal immune activation produces cerebellar hyperplasia and alterations in motor and social behaviors in male and female mice. Cerebellum.

[32] Hsiao E.Y., McBride S.W., Hsien S., Sharon G., Hyde E.R., McCue T., Codelli J.A., Chow J., Reisman S.E., Petrosino J.F. (2013). Microbiota Modulate Behavioral and Physiological Abnormalities Associated with Neurodevelopmental Disorders. Cell.

[33] Malkova N.V., Yu C.Z., Hsiao E.Y., Moore M.J., Patterson P.H. (2012). Maternal immune activation yields offspring displaying mouse versions of the three core symptoms of autism. Brain Behav. Immun..

[34] Lins B.R., Hurtubise J.L., Roebuck A.J., Marks W.N., Zabder N.K., Scott G.A., Greba Q., Dawicki W., Zhang X., Rudulier C.D. (2018). Prospective Analysis of the Effects of Maternal Immune Activation on Rat Cytokines during Pregnancy and Behavior of the Male Offspring Relevant to Schizophrenia. eNeuro.

[35] Vigli D., Palombelli G., Fanelli S., Calamandrei G., Canese R., Mosca L., Scattoni M.L., Ricceri L. (2020). Maternal Immune Activation in Mice Only Partially Recapitulates the Autism Spectrum Disorders Symptomatology. Neuroscience.

[36] Gray A., Tattoli R., Dunn A., Hodgson D.M., Michie P.T., Harms L. (2019). Maternal immune activation in mid-late gestation alters amphetamine sensitivity and object recognition, but not other schizophrenia-related behaviours in adult rats. Behav. Brain Res..

[37] Ruskin D.N., Murphy M.I., Slade S.L., Masino S.A. (2017). Ketogenic diet improves behaviors in a maternal immune activation model of autism spectrum disorder. PLoS ONE.

[38] Kirsten T.B., Taricano M., Maiorka P.C., Palermo-Neto J., Bernardi M.M. (2010). Prenatal lipopolysaccharide reduces social behavior in male offspring. Neuroimmunomodulation.

[39] Taylor P.V., Veenema A.H., Paul M.J., Bredewold R., Isaacs S., de Vries G.J. (2012). Sexually dimorphic effects of a prenatal immune challenge on social play and vasopressin expression in juvenile rats. Biol. Sex Differ..

[40] Carlezon W.A., Kim W., Missig G., Finger B.C., Landino S.M., Alexander A.J., Mokler E.L., Robbins J.O., Li Y., Bolshakov V.Y. (2019). Maternal and early postnatal immune activation produce sex-specific effects on autism-like behaviors and neuroimmune function in mice. Sci. Rep..

[41] Morais L.H., Felice D., Golubeva A.V., Moloney G., Dinan T.G., Cryan J.F. (2018). Strain differences in the susceptibility to the gut-brain axis and neurobehavioural alterations induced by maternal immune activation in mice. Behav. Pharmacol..

[42] Cieslik M., Gassowska-Dobrowolska M., Jesko H., Czapski G.A., Wilkaniec A., Zawadzka A., Dominiak A., Polowy R., Filipkowski R.K., Boguszewski P.M. (2020). Maternal Immune Activation Induces Neuroinflammation and Cortical Synaptic Deficits in the Adolescent Rat Offspring. Int. J. Mol. Sci..

[43] Knutson B., Burgdorf J., Panksepp J. (1998). Anticipation of play elicits high-frequency ultrasonic vocalizations in young rats. J. Comp. Psychol..

[44] Himmler B.T., Kisko T.M., Euston D.R., Kolb B., Pellis S.M. (2014). Are 50-kHz calls used as play signals in the playful interactions of rats? I. Evidence from the timing and context of their use. Behav. Process..

[45] Burke C.J., Kisko T.M., Swiftwolfe H., Pellis S.M., Euston D.R. (2017). Specific 50-kHz vocalizations are tightly linked to particular types of behavior in juvenile rats anticipating play. PLoS ONE.

[46] Mulvihill K.G., Brudzynski S.M. (2018). Non-pharmacological induction of rat 50 kHz ultrasonic vocalization: Social and non-social contexts differentially induce 50 kHz call subtypes. Physiol. Behav..

[47] Baum S.H., Stevenson R.A., Wallace M.T. (2015). Behavioral, perceptual, and neural alterations in sensory and multisensory function in autism spectrum disorder. Prog. Neurobiol..

[48] Tschida K., Michael V., Takatoh J., Han B.-X., Zhao S., Sakurai K., Mooney R., Wang F. (2019). A Specialized Neural Circuit Gates Social Vocalizations in the Mouse. Neuron.

[49] Kelm-Nelson C.A., Gammie S. (2020). Gene expression within the periaqueductal gray is linked to vocal behavior and early-onset parkinsonism in Pink1 knockout rats. BMC Genom..

[50] Melancia F., Schiavi S., Servadio M., Cartocci V., Campolongo P., Palmery M., Pallottini V., Trezza V. (2018). Sex-specific autistic endophenotypes induced by prenatal exposure to valproic acid involve anandamide signalling. Br. J. Pharmacol..

