# Supplementary material for: The Effect of Maternal Immune Activation on Social Play-Induced Ultrasonic Vocalization in Rats
Source: Brain Sci. 2021 Mar 9;11(3):344. doi: 10.3390/brainsci11030344 (PMC8001568; doi:10.3390/brainsci11030344)
Supplement: Supplementary file 1 [file brainsci-11-00344-s001.pdf]

Supplement 1. Rats' body weight.

There were no differences between either vehicle- and poly(I:C)-treated rats or males and females.

Figure S1. Body weight.

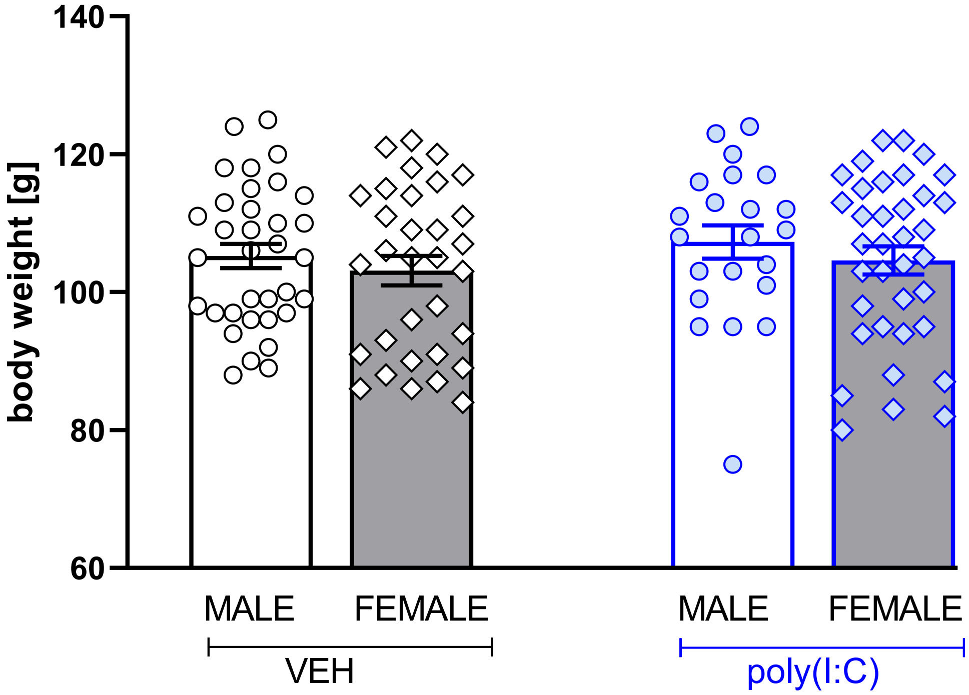

The number of animals used in the analysis was: N=34 (vehicle males), N=32 (vehicle females), N=22 (poly(I:C) males), and N=36 (poly(I:C) females).

Table S1. Body weight - ANOVA results.

Effect		Body weight		
	df	F	p	η_p^2
**Treatment (T)**	1,120	0.876	0.351	0.009
**Sex (S)**	1,120	1.044	0.309	0.007
**T×S**	1,120	0.061	0.805	0.001

Supplement 2. **Statistics.**

Table S2. The results of the ANOVA analysis.

* In case data were not normally distributed (i.e., pinning episodes, latency to pinning, play responsiveness, and marble burying test), differences between groups were analyzed using Mann-Whitney U-test.

Social play behavior							
Effect	df	pouncing			Total play duration		
		F	p	η_p^2	F	p	η_p^2
Treatment (T)	1,58	0.205	0.653	0.004	1.516	0.223	0.025
Sex (S)	1,58	1.022	0.316	0.017	1.738	0.193	0.029
T×S	1,58	1.086	0.302	0.018	6.953	0.011	0.107

Ultrasonic vocalizations													
Effect	df	USV number			duration			bandwidth			peak frequency		
		F	p	η_p^2	F	p	η_p^2	F	p	η_p^2	F	p	η_p^2
Treatment (T)	1,58	8.317	0.005	0.125	0.900	0.347	0.015	0.114	0.737	0.002	4.324	0.042	0.069
Sex (S)	1,58	11.49	0.001	0.165	10.12	0.002	0.149	6.278	0.015	0.098	1.649	0.206	0.027
T×S	1,58	0.963	0.330	0.016	0.071	0.793	0.001	0.201	0.655	0.003	0.292	0.569	0.005

percentage USV distribution				
	df	F	p	η_p^2
call types (C)	4,232	124.39	<0.001	0.682
C×T	4,232	2.963	0.021	0.049
C×S	4,232	5.035	<0.001	0.079
C×T×S	4,232	0.244	0.677	0.004

Locomotor and repetitive/stereotypic-like activity										
Effect	df1	df2	Distance traveled			df1	df2	Stereotypic movements		
			F	p	η_p^2			F	p	η_p^2
**Treatment (T)**	1	69	5.913	0.017	0.079	1	69	12.474	<0.001	0.153
**Sex (S)**	1	69	29.949	<0.001	0.03	1	69	0.125	0.725	0.002
**T×S**	1	69	0.358	0.552	0.005	1	69	0.002	0.969	0.000
**Time**	3.92	234.11	94.014	<0.001	0.577	2.74	189.08	5.594	0.002	0.075
**Time×T**	3.92	234.11	0.785	0.517	0.011	2.74	189.08	0.997	0.391	0.014
**Time×S**	3.92	234.11	3.503	0.012	0.048	2.74	189.08	0.494	0.672	0.007
**Time×T×S**	3.92	234.11	0.646	0.605	0.009	2.74	189.08	0.696	0.543	0.009

marble burying test – distance traveled				
	df	F	p	η_p^2
**Treatment (T)**	70	1.285	0.261	0.018
**Sex (S)**	70	0.268	0.606	0.004
**T×S**	70	0.044	0.834	0.001

Supplement 3. Trill calls.

Poly (I:C) treatment did not affect the acoustic parameters of trill calls. The sex-differences were demonstrated in the duration measure, as the trills emitted by females were shorter than in males (p<0.01, Tukey HSD post hoc test following a significant sex effect).

Figure S2. The acoustic characteristics of trill calls.

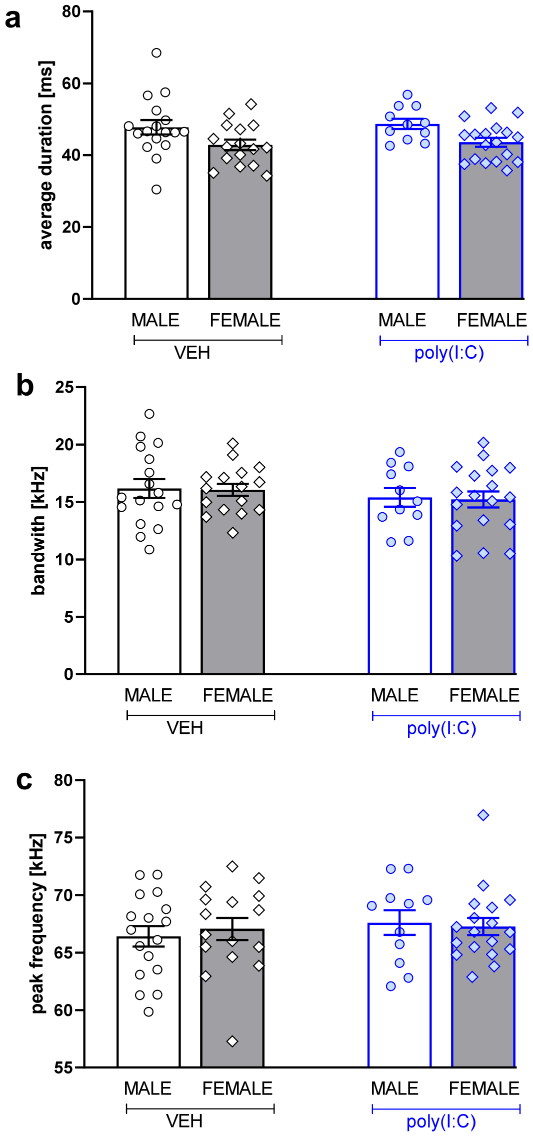

Table S3. The acoustic characteristics of trill calls – ANOVA results.

Anova							
Effect		duration		bandwidth		peak frequency	
	df	F	p	F	p	F	p
Treatment (T)	1,59	0.207	0.608	1.211	0.276	0.584	0.449
Sex (S)	1,59	9.485	0.003	0.018	0.895	0.028	0.867
T×S	1,59	0.005	0.992	0.002	0.968	0.286	0.595

Supplement 4. Circling behavior.

Figure S3. Poly (I:C) treatment increased circling behaviors in females.

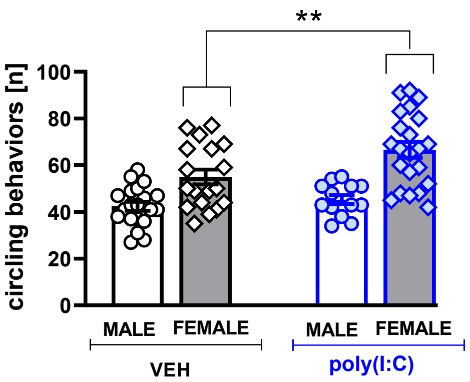

Data are presented as a mean ± SEM of the total number of clockwise and counter-clockwise rotations in auto-track cages during a 30-min session. Symbols: **p<0.01, a significant difference between vehicle- and poly(I:C)-exposed animals in a given sex group (Student's t-test).

circling behaviors				
	**df**	**F**	**p**	**η_p^2**
**Treatment (T)**	69	6.104	0.0159	0.0812
**Sex (S)**	69	33.598	<0.001	0.327
**T×S**	69	2.254	0.138	0.032
